# Assessing the Concordance Between Urogenital and Vaginal Microbiota: Can Urine Specimens Be Used as a Proxy for Vaginal Samples?

**DOI:** 10.3389/fcimb.2021.671413

**Published:** 2021-06-29

**Authors:** Sarah E. Brown, Courtney K. Robinson, Michelle D. Shardell, Johanna B. Holm, Jacques Ravel, Khalil G. Ghanem, Rebecca M. Brotman

**Affiliations:** ^1^ Institute for Genome Sciences, University of Maryland School of Medicine, Baltimore, MD, United States; ^2^ Department of Epidemiology and Public Health, University of Maryland School of Medicine, Baltimore, MD, United States; ^3^ Department of Microbiology and Immunology, University of Maryland School of Medicine, Baltimore, MD, United States; ^4^ Division of Infectious Diseases, Johns Hopkins University School of Medicine, Baltimore, MD, United States

**Keywords:** 16S rRNA gene amplicon sequencing, clean-catch urine, random-catch urine, vaginal microbiota, community state type

## Abstract

**Background:**

The vaginal microbiota play a key role in defense against reproductive tract infections; however, many population-based women’s health studies do not collect vaginal samples. Molecular examinations of urine samples have revealed common vaginal bacteria. We sought to assess the extent that community state type assignments of archived random-catch and clean-catch urine samples agreed with the paired vaginal samples in both reproductive-age and peri/post-menopausal women.

**Results:**

Using archived samples, we evaluated the microbiota concordance among women in three studies: two with paired mid-vaginal/random-catch urine (N=91 reproductive-age participants and N=13 peri/post-menopausal participants), and one with paired mid-vaginal/clean-catch urine (N=99 reproductive-age participants). Microbiota composition was characterized by sequencing amplicons of the 16S rRNA gene V3-V4 regions and assigned to community state types. Similarity of paired samples was gauged using agreement of community state types and Yue-Clayton θ indices. Analysis of Composition of Microbiomes II indicated which taxa were differently relatively abundant in paired vaginal and urine samples. In reproductive-age women, random-catch and clean-catch urines were 89.0% and 86.9% concordant on five community state types with paired mid-vaginal swabs, and Kappa statistics indicated almost perfect agreement (κ_random-catch_=.85, κ_clean-catch_=.81, p<0.0001). A small number of pairs of samples were discordant (23/190, 12%), and discordant pairs tended to be between samples classified to *L. iners*-dominated and/or low-*Lactobacillus* states. Concordance and agreement remained similar when dichotomizing the microbiota to *Lactobacillus-*dominated *versus* low-*Lactobacillus* microbiota, as well as when evaluating separately the three subtypes of the low-*Lactobacillus* community state type IV. Median similarity of paired urine/vaginal samples was high (θ_random-catch_=.85, θ_clean-catch_=.88), and a comparison of the random-catch and clean-catch similarity scores showed no significant difference (p=.80). Concordance and similarity were lower for peri/post-menopausal women, but agreement remained substantial (76.9% concordant, κ_random-catch_= 0.64, θ_random-catch_=.62). Taxonomic-level analysis confirmed these findings.

**Conclusions:**

Random-catch and clean-catch urine samples showed substantial agreement on bacterial composition to paired mid-vaginal samples, indicating that the genitourinary microbiota may be a reliable proxy for assessing the overall composition of the vaginal microbiota *via* community state types. This data suggests that urine samples can, with proper interpretation, be utilized as a surrogate for developing preliminary data and hypothesis-generating studies.

## Introduction

The composition of the vaginal microbiota has critical implications for susceptibility to sexually transmitted infections (STIs), miscarriage, and spontaneous preterm delivery (Martin, 2012; Aldunate et al., 2015; Gosmann et al., 2017; Klatt et al., 2017; Tyssen et al., 2018; Elovitz et al., 2019; Al-Memar et al., 2020). The mechanism is in part attributed to the action of *Lactobacillus* spp., many of which provide broad-spectrum protection *via* lactic acid (Boskey et al., 2001; Aldunate et al., 2013; Tyssen et al., 2018; Edwards et al., 2019). Recent studies applying culture-independent methods have allowed for the detection of a quantifiable and diverse urinary microbiota (Nelson et al., 2010; Dong et al., 2011; Wolfe et al., 2012; Pearce et al., 2014; Mueller et al., 2017; Adebayo et al., 2020), and these findings have been validated with quantitative enhanced culture methods (Hilt et al., 2014; Coorevits et al., 2017).

Several organisms commonly found in the vagina have been observed in urine samples (Siddiqui et al., 2011; Pearce et al., 2014; Thomas-White et al., 2017), and bacterial strains isolated from the bladder and vagina have been found to be functionally and phylogenetically similar (Thomas-White et al., 2018a). In one study, voided urine samples demonstrated more similarity to paired vaginal swabs than to paired supra-pubic needle aspirates or trans-urethral catheterized samples (Wolfe et al., 2012). This suggests that the microbiota of some types of urine samples may more closely resemble vaginal microbiota than other urine sample types; however, there is also similarity at the genus-level between paired vaginal and trans-urethral catheterized samples (Komesu et al., 2019). Given the overlap between the genitourinary and vaginal microbiota, we hypothesized that voided urine may be used as a proxy for vaginal community assessment in research studies utilizing 16S rRNA gene amplicon sequencing.

To evaluate the use of urine as a proxy for vaginal swabs, we sought to compare the microbiota of paired mid-vaginal swabs with the microbiota of urine samples collected using “clean-catch” (CC) or “random-catch” (RC) methods from reproductive-aged women, and paired mid-vaginal swabs and RC urine samples from peri/post-menopausal women. The first-void of the initial urine stream is collected for RC urine samples while, for urine collected *via* the CC method, the labia are cleaned with an antibacterial wipe and mid-stream urine is collected. While the microbiota of both RC and CC urine samples might be similar in composition to vaginal microbiota because of shared species, RC urine may contain a higher proportion of vulvovaginal bacteria due to contamination from the urine stream washing over the labia, resulting in a better proxy of the vaginal microbiota than CC urine.

To our knowledge, CC and RC urine samples have not been assessed in conjunction with the vaginal microbiota and, although the concordance between the urinary and vaginal microbiota of peri/post-menopausal women has been studied (Komesu et al., 2019), they have not been evaluated separately from reproductive-age women. Peri/post-menopausal women have different vaginal (Brotman et al., 2014; Gliniewicz et al., 2019; Shardell et al., 2021) and urinary (Ammitzbøll et al., 2021) microbiota compared to reproductive-age women, and may carry lower bacterial loads (Holm et al., 2019). These differences may affect the extent to which the genitourinary and vaginal microbiota overlap. We aim to fill this knowledge gap by determining whether concordance data support the use of voided urine specimens as a proxy for broadly assessing the composition of the vaginal microbiota.

## Materials and Methods

### Sample Collection

We utilized archived mid-vaginal swabs and urine samples collected at the same study visit from reproductive-age and peri/post-menopausal women who had enrolled in three separate observational cohort studies. All women contributed a single observation day to the dataset.

First, RC urine samples and paired mid-vaginal swabs were collected from 113 reproductive-age participants enrolled in a study of nonpregnant women at the University of Alabama at Birmingham (ages 18 to 45) as previously described (Ravel et al., 2013). Participants were asked to self-collect a mid-vaginal Copan ESwab (Copan Diagnostics) that was stored in Amies liquid transport medium and collect a first-void urine sample. Urine samples were aliquoted and frozen within one hour of collection, and all samples were stored at -80°C until DNA extraction.

Second, RC urine samples and paired mid-vaginal swabs were collected from 15 peri/post-menopausal participants enrolled in the Gynecology and Lubricant Effects Study at the University of Maryland School of Medicine (ages 44 to 69 years) as previously described (Crowder et al., 2019). Participants were asked to self-collect a mid-vaginal Copan Eswab (Copan Diagnostics) that was stored in equal parts Amies liquid transport medium and RNAlater (ID 230 only) or modified C2 (MoBio), and collect a first-void urine sample. Urine samples were aliquoted and refrigerated within 1 hour, and frozen within 24 hours of collection, and all samples were stored at -80°C until DNA extraction.

Third, CC urine samples and paired mid-vaginal swabs were collected from 123 reproductive-age participants enrolled in the Hormonal Contraceptives Longitudinal Study, a cohort study recruited at the Johns Hopkins University School of Medicine, Baltimore, MD (ages 16 to 35 years), as previously described (Tuddenham et al., 2019). Participants were asked to self-collect a mid-vaginal Copan ESwab (Copan Diagnostics) that was stored in Amies liquid transport medium. For the urine sample, participants were asked to wipe the labia with a standard wipe containing chlorhexidine and collect a mid-stream specimen. Urine samples were aliquoted and frozen within one hour of collection, and all samples were stored at -80°C until DNA extraction.

All participants provided informed consent. The Institutional Review Boards at the University of Alabama Birmingham, Johns Hopkins University School of Medicine and the University of Maryland School of Medicine approved the protocols.

### Genomic DNA Extractions

Genomic DNA was extracted from mid-vaginal swabs with either the QS DSP Virus/Pathogen Midi Kit (Qiagen, Germantown, MD) on the QiaSymphony platform or with the MagAttract Microbial DNA Kit (Qiagen, Germantown, MD) using a custom automated protocol on the Hamilton Microlab STAR **(** **Table S4** **)**. For the QiaSymphony platform, vaginal swabs were thawed on ice and 500 µL of the Amies transport medium was used as input following the protocol described in Holm et al. (2019). For the MagAttract kit, swabs were thawed on ice and a 200 µL aliquot from the Amies transport medium was used as input for the kit following the manufacturer protocol adapted for use on the Hamilton STAR robot. Cells were lysed *via* shaking on a TissueLyser (Qiagen, Germantown, MD) at 20Hz for 20 min. Negative controls consisting of 200 µL distilled sterile water were extracted in the same manner as samples.

DNA extractions on the RC urine samples from reproductive-age participants were performed on 1.5 mL urine using the Quick-DNA Urine Kit (Zymo Research, Irvine, CA). After addition of the Urine conditioning buffer and clearing beads, pelleted cells were resuspended in 720 µL of Lysis solution. Two enzymatic lysis steps were performed: 1) 40 µg Lysozyme, 120 U Mutanolysin, and 2.5 µg Lysostaphin were added, and samples were incubated at 37°C for 30 min and 2) 160 µg Proteinase K, 0.5% (final concentration) SDS, and 16 µg RNase was added and samples were incubated at 55°C for 45 min. A mechanical lysis step was performed with samples in Lysing Matrix B (MPBio) tubes and processed at 6 m/s for 40 s in the FastPrep. Subsequent washing steps were performed according to the Quick-DNA Urine kit manufacturer’s standard protocol.

To test the feasibility of DNA extractions on urine in a more high-throughput manner, extractions on the CC urine samples from reproductive-age participants, and on the RC urine samples from peri/post-menopausal participants were performed with a hybrid approach, starting with the Quick-DNA Urine kit and ending with the MagAttract Microbial DNA Kit (Qiagen, Germantown, MD). Briefly, a 1 mL aliquot of urine was mixed with 70 µL of Urine Conditioning Buffer and 10 µL of Clearing Beads, both from the Quick-DNA Urine kit, and centrifuged at 3,000 x *g* for 15 min. The resulting pellet was resuspended in 650 µL of the Lysis buffer from the MagAttract kit and samples were transferred to a PowerBead DNA plate containing 0.1 mm glass beads. The rest of the extraction was performed with the MagAttract kit following a protocol adapted for use on the Hamilton STAR robot.

### Library Construction and Sequencing

Relative abundances of bacteria were assessed through amplification and sequencing of the 16S rRNA gene V3-V4 regions. PCR amplifications were carried out in either 1- or 2-step reactions and sequenced on the MiSeq or HiSeq platforms (Illumina, San Diego, CA) as described in Holm et al. **(** **Table S4** **)** (Holm et al., 2019).

### Post-Sequencing Data Processing, Taxonomy and CST Assignments

Post-sequencing data processing was done separately for samples from reproductive-age and peri/post-menopausal participants. Sequence de-multiplexing, removal of barcode sequences, and further sample processing were carried out with QIIME-dependent scripts, TagCleaner, and the DADA2 Workflow for Big Data as previously described (Holm et al., 2019). Amplicon sequence variants (ASVs) generated by DADA2 were classified using the RDP Naïve Bayesian Classifier (Wang et al., 2007) trained with the SILVA v128 16S rRNA gene sequence database (Quast et al., 2013) as implemented in the *dada2* R package (Callahan et al., 2016). ASVs of major vaginal taxa were assigned species-level annotations using *speciateIT* (http://ravel-lab.org/speciateit/). Extraction and PCR negative controls were examined for contaminating taxa, and all reads from contaminating taxa were removed from the overall dataset (*Pseudomonas* and *Achromobacter* in samples from reproductive-age women, and none in samples from peri/post-menopausal women). Taxa present at less than 10^-5^ across all samples were removed and samples with fewer than 500 reads were removed from analysis.

Community state types (CSTs) were assigned using VALENCIA, an algorithm based on similarity to the centroid of each cluster (France et al., 2020).

### Statistical Analysis

Community similarity at the CST-level was compared with Cohen’s kappa coefficient as implemented in SAS Studio Version 3.8. Seven, five and two levels of CST were assessed. Similarity of microbial populations from paired vaginal swabs and urine samples was computed with Yue-Clayton θ indices, which take into account the relative abundances of shared and non-shared species in each population (Yue and Clayton, 2005), and a Wilcoxon rank sum test was used to compare the distributions of similarity indices of paired mid-vaginal/RC urine samples to paired mid-vaginal/CC urine samples. Among reproductive-age women, ANCOM II was used to detect taxa with significantly different relative abundances comparing paired vaginal swabs and urine samples, accounting for random subject effects and adjusting for urine sample type (Kaul et al., 2017). The ANCOM II analysis was carried out using the script ANCOM v2.1 (https://github.com/FrederickHuangLin/ANCOM) in R Studio Version 1.0.143.

## Results

Demographic, health, and behavioral characteristics of participants are given in **Table 1**. Across all studies, amplicons sequencing yielded 25,592,992 high-quality sequences. After removing potential contaminants, low-abundant taxa, and samples with low read counts, 91 (80.5%) and 13 (86.7%) pairs of mid-vaginal/RC urine samples were retained for reproductive-age and peri/post-menopausal women, respectively (**Table S1**). Of the sample pairs dropped, 21 pairs of mid-vaginal/RC urine samples from reproductive-age women and 2 pairs of mid-vaginal/RC urine samples from peri/post-menopausal women were excluded because of low sequencing counts in the vaginal samples. A total of 99 pairs of mid-vaginal/CC urine samples (80.5%) were retained for analysis, and 24 pairs were dropped because of low sequence counts in the urine samples.

**Table 1:** Demographic, health, and behavioral characteristics of participants in each study.

	Project		
	Reproductive-age women with random-catch urine (N=91)	Reproductive-age women with clean-catch urine (N=99)	Peri/post-menopausal women with random-catch urine (N=13)
	*N (%) Mean (SD)*	*N (%) Mean (SD)*	*N (%) Mean (SD)*
Age (years)	28 (6.4)	26 (4.3)	55 (6.4)
Race			
Black	59 (65)	33 (35)	9 (69)
White	27 (30)	57 (58)	1 (8)
Multiracial	1 (1)	6 (6)	0 (0)
Other	4 (4)	3 (3)	3 (23)
Ever had vaginal sex	85 (93)	95 (96)	13 (100)
Currently using hormonal contraception^1^	26 (29)	44 (44)	0 (0)
Currently experiencing menses or vaginal bleeding	10 (11)	7 (7)	1 (9)
Clinical findings			
None	63 (69)	87 (88)	10 (77)
Asymptomatic bacterial vaginosis	23 (25)	2 (2)	2 (15)
Symptomatic bacterial vaginosis	0 (0)	2 (2)	0 (0)
Yeast infection	3 (3)	1 (1)	0 (0)
Other	2 (2)	3 (3)	1 (8)
Missing	0 (0)	4 (4)	0 (0)

^1^Includes oral contraceptive pill, progesterone-containing intrauterine device, implant, injection, and patch.

In the vaginal microbiota, CSTs may be dominated by a few specific organisms (Ravel et al., 2011; Gosmann et al., 2017; Mckinnon et al., 2019). VALENCIA identified five CSTs in the urine and vaginal samples, four of which were dominated by the indicated *Lactobacillus* species: CST I: *Lactobacillus crispatus*, CST II: *L. gasseri*, CST III: *L. iners*, and CST V: *L. jensenii*. CST IV is a low-*Lactobacillus* state and could be further divided into three sub-CSTs based on the most abundant organisms detected (CST IV-A: “*Ca. Lachnocurva vaginae”* and *Gardnerella*, CST IV-B: *Gardnerella*, *Sneathia sanguinegens*, and *Atopobium vaginae*, and CST IV-C: *Streptococcus* spp. and *Corynebacterium*).

**Figure 1:**
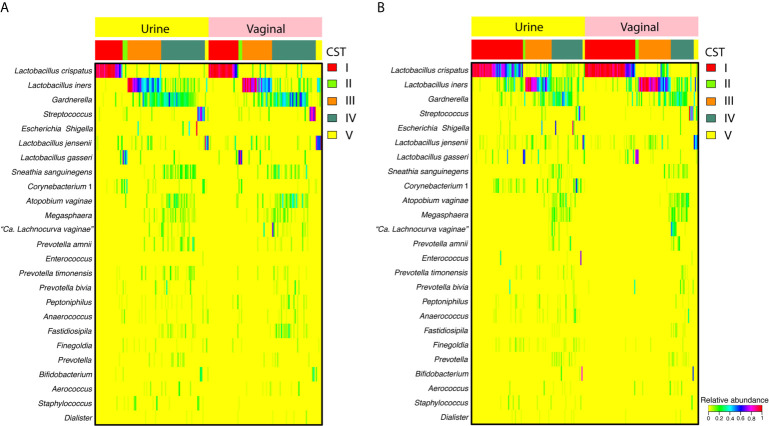
Heatmap displaying the relative abundance of the 25 most abundant taxa in random-catch **(A)** or clean-catch **(B)** urine samples and paired vaginal swabs. Community state types (CSTs) are indicated in the second row from the top.

Similar taxonomic compositions and overall community structures were apparent when comparing paired mid-vaginal/RC urine samples and paired mid-vaginal/CC urine samples in reproductive-age women **(** **Figure 1** **)**. Principle coordinate analysis demonstrated that paired mid-vaginal/RC urine and mid-vaginal/CC urine samples clustered by CST, rather than sample type **(** **Figure S1** **)**.

**Figure 2:**
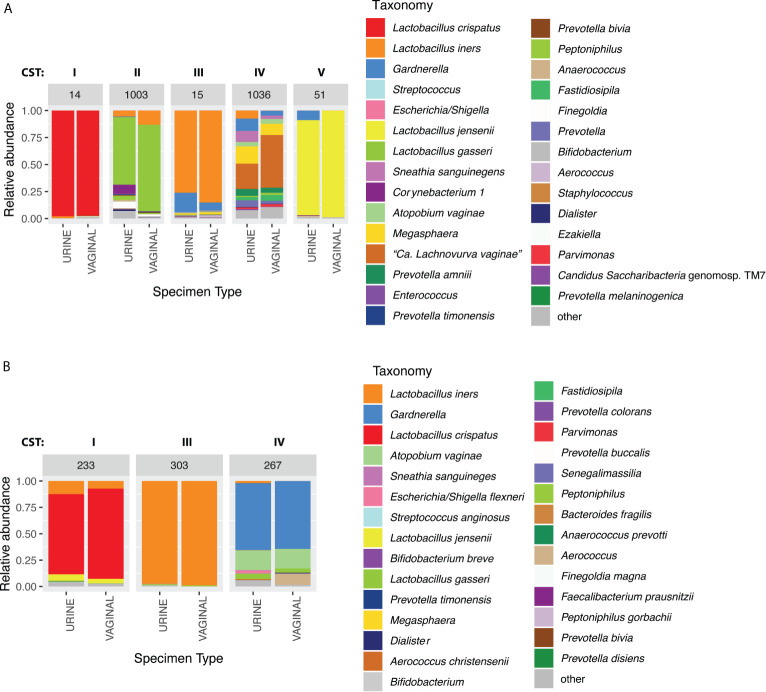
Example taxonomic composition for paired urine and vaginal samples that were concordant on CST in **(A)** reproductive-age women (paired clean-catch urine IDs: 1003, 1036. Paired random-catch urine IDs: 14, 15, 51) and **(B)** peri/post-menopausal women (all random-catch urine). Assigned CSTs are indicated above each sample pair.

In reproductive-age women, there was an 89.0% concordance in 5-level CST between paired mid-vaginal/RC urine samples **(** **Table 2** **)** and an 86.9% concordance between paired mid-vaginal/CC urine samples (**Table 3**). Kappa statistics indicated almost perfect chance-adjusted agreement (**Table 4**, κ_RC_=0.85, 95% CI=0.75-0.94, κ_CC_=0.81, 95% CI=0.71-0.90). In peri/post menopausal women, there was a 76.9% concordance in 5-level CST between paired mid-vaginal/RC urine samples (**Table 2**), and the Kappa statistic indicated substantial chance-adjusted agreement (**Table 4**, κ_RC_=0.64, 95% CI=0.31-0.97). **Figure 2** presents stacked taxonomic relative abundance plots of CST-concordant pairs; similar community structures were often observed between sample types.

**Table 2:** Concordance of random-catch urine to paired vaginal samples in reproductive-age (N=91) and peri/post-menopausal (N=13) women.

	Vaginal CST Reproductive-age women					Vaginal CST Peri/post-menopausal women				
	I	II	III	IV	V	I	II	III	IV	V
**Urine CST**										
**I**	**22**	0	0	0	0	**1**	0	0	0	0
**II**	1	**3**	0	0	0	0	**0**	0	0	0
**III**	1	0	**21**	3	2	0	0	**3**	0	0
**IV**	0	0	3	**32**	0	0	0	0	**6**	1
**V**	0	0	0	0	**3**	0	1	0	1	**0**

**Table 3:** Concordance of clean-catch urine with paired vaginal samples in reproductive-age women (N=99).

	Vaginal CST				
	I	II	III	IV	V
**Urine CST**					
**I**	**41**	0	4	0	0
**II**	0	**2**	0	0	0
**III**	1	1	**21**	0	0
**IV**	2	0	3	**20**	2
**V**	0	0	0	0	**2**

**Table 4:** Agreement between urogenital and vaginal microbiota composition measured by Cohen’s kappa statistic.

Categorical Analysis	Group	Urine Sample	κ	95% CI	p-value
5 levels:	Reproductive-age	RC	0.85	0.75-0.94	<0.0001
CSTs I, II, III, IV, V	Reproductive-age	CC	0.81	0.71-0.90	<0.0001
	Peri/post-menopausal	RC	0.64	0.31-0.97	<0.001
7 levels:	Reproductive-age	RC	0.77	0.67-0.87	<0.0001
CSTs I, II, III, IV-A, IV-B, IV-C, V	Reproductive-age	CC	0.74	0.64-0.85	<0.0001

	Peri/post-menopausal	RC	0.69	0.41-0.97	<0.0001
2 levels:	Reproductive-age	RC	0.86	0.75-0.97	<0.0001
*Lactobacillus-*dominated and low-*Lactobacillus*	Reproductive-age	CC	0.81	0.67-0.94	<0.001
	Peri/post-menopausal	RC	0.69	0.30-1.00	0.01

In reproductive-age women, 11% (10/91) and 13.1% (13/99) of paired mid-vaginal/RC urine and mid-vaginal/CC urine samples were discordant for CST, respectively (**Figure S2**). The most common disagreement was between CSTs IV and III (9/23), and differences in the proportion of *L. iners* often accounted for the discordance (**Figure S2**). Other samples discordant for CST also shared similar community structures overall. In peri/post-menopausal women, 23.1% (3/13) of the paired mid-vaginal/RC urine samples were discordant for CST **(** **Figure S3** **)**.

We carried out a sensitivity analysis to determine the robustness of urine to discriminate between CSTs when the three sub-CSTs of CST IV were identified. Paired mid-vaginal/RC urine samples were 82.4% concordant **(** **Table S2** **)**, and paired >mid-vaginal/CC urine samples were 81.8% concordant (**Table S3**), with Kappa indicating substantial chance-adjusted agreement for both in reproductive-age women (**Table 4**, κ_RC_=0.77, 95% CI=0.67-0.87, κ_CC_=0.74, 95% CI=0.64-0.90). Paired mid-vaginal/RC urine samples in peri/post-menopausal women were 76.9% concordant (**Table S2**), with Kappa indicating substantial chance-adjusted agreement (**Table 4**, κ_RC_=0.69, 95% CI=0.41-0.97).

We also evaluated how well urine samples broadly discriminate between communities dominated by *Lactobacillus* spp. *versus* communities with low levels of *Lactobacillus* spp. When CSTs I, II, III, and V were consolidated into a *Lactobacillus*-dominated group and CST IV was considered non-*Lactobacillus* dominated, the concordance for paired mid-vaginal/RC urine samples in reproductive-age and peri/post-menopausal women increased to 93.4% and 84.6%, respectively (**Table S4**, κ_RC_=0.86, 95% CI=0.75-0.97, and κ_RC_=0.69, 95% CI=0.30-1.00). The concordance for paired mid-vaginal/CC urine samples in reproductive-age women increased to 92.9% (**Table S5**, κ_CC_=0.81, 95% CI=0.67-0.94).

The community-level similarities between paired mid-vaginal/urine samples were also estimated with the Yue-Clayton θ index (Yue and Clayton, 2005). The median similarity of RC and CC urine to paired mid-vaginal swabs indicates a high degree of similarity in reproductive-age women (θ_RC_=.85 and θ_CC_=.88), and a moderate degree of similarity in peri/post-menopausal women (θ_RC_=.62). Comparison of the distribution of θ similarity scores for paired mid-vaginal/RC urine samples to mid-vaginal/CC urine samples in reproductive-age women showed no significant difference (p=0.80).

**Figure S4A** shows the taxa identified by ANCOM II as being most likely to be different in their relative abundance comparing paired vaginal and urine samples, For each taxa, the W value represents the number of log-ratio hypothesis tests in which the ratio between that taxa and another taxa was significantly different in vaginal *versus* urine samples, with higher W values indicating stronger statistical evidence. The recommended cutoff for significance is a W value above the 70^th^ percentile, although higher percentile cutoffs can be used for more conservative results and to decrease the false discovery rate. Ten of the top 25 most abundant taxa in reproductive-age women had W values above the 70^th^ percentile, indicating their relative abundance was more likely to be differ by sample type compared to the relative abundance of other taxa. **Figure S4B** shows the distribution of paired differences in relative abundance (% abundance vaginal - % abundance urine) for the 10 most abundant taxa identified by ANCOM II. For each taxa, the median paired difference in relative abundance was approximately 0%. Mean paired differences in relative abundance ranged from 2.7% higher in urine samples (*Corynebacterium* 1) to 0.9% higher in vaginal samples (*Streptococcus*).

## Discussion

The vaginal microbiota play a major role in sexual and reproductive health, and ongoing studies continue to explore the mechanisms of protection and interventions to optimize the vaginal microbiome. However, not all women’s health studies, particularly large population-based studies, include vaginal sampling. We assessed whether urine samples could serve as a proxy for vaginal samples in studies utilizing 16S rRNA gene amplicon sequencing to determine community state type (CST). We evaluated whether the CST assigned to a RC or CC urine sample was concordant with the CST assigned to the paired vaginal swab, and whether compositional measurements also indicated overall community similarity.

### CST Concordance

For reproductive-age and peri/post-menopausal women, we found substantial agreement in the composition of the microbiota between both RC and CC urine samples and paired mid-vaginal swabs. This was true when comparing compositional similarity, when dichotomizing a woman’s genitourinary microbiota to either *Lactobacillus-*dominated or low-*Lactobacillus*, as well as for higher resolution analyses using 5- or 7-level categorical CSTs. Importantly, clustering by CST allows identification of vaginal microbiota dominated by *L. iners* (CST III), which is often found in the vaginal microbiota of women with bacterial vaginosis (BV) (Zozaya-Hinchliffe et al., 2010; Srinivasan et al., 2012; Petrova et al., 2017). As we learn more about the functional and genomic diversity of *L. iners* (Macklaim et al., 2013), it is becoming evident that grouping all *L. iners*-dominated vaginal microbiota with other *Lactobacillus*-dominated CSTs may not be appropriate as it might not confer the same protection against adverse reproductive health outcomes (Van Houdt et al., 2018; Sarmento et al., 2021).

It is not entirely surprising that there is a high degree of similarity between the composition of the vaginal and genitourinary microbiota using CSTs. As previously mentioned, voided urine, particularly random-catch urine, is likely to be contaminated with vulvovaginal bacteria. Similar to our findings, a 2014 study also reported no significant difference in the accuracy of diagnosing BV using species-specific qPCR methods when comparing random-catch (first void) urine samples to paired vaginal swabs (Datcu et al., 2014). There are also demonstrated parallels between the vaginal microbiota and the microbiota of the bladder and lower urinary tract. One study found post-operative UTI was less likely among women with *Lactobacillus*, particularly *L. iners*, detected in their pre-operative urinary microbiota (Thomas-White et al., 2018b). This finding seems to suggest that, similar to what is seen in the vaginal microbiota with *Lactobacillus* spp. and reproductive tract infections, *Lactobacillus* spp. in the bladder may be associated with decreased risk of urinary tract infections, although more studies are needed to confirm these findings. Price et al. reported, as with the vaginal microbiota (Gajer et al., 2012), the composition of the lower urinary tract microbiota is also affected by sexual behaviors and menstruation (Price et al., 2020).

In menopause, urogenital tissues are affected by decreasing estrogen levels and are associated with recurrent UTIs, increased urinary frequency, genital dryness, thinning of the vaginal epithelium, and a loss of vaginal *Lactobacillus* spp. as well as a decrease in vaginal bacterial load (Hillier and Lau, 1997; Portman et al., 2014; Holm et al., 2019). These signs and symptoms, along with others, define the genitourinary syndrome of menopause (GSM) – a condition which affects approximately half of post-menopausal women (Portman et al., 2014). Because GSM affects both the vaginal microbiota and urinary tract (Thomas-White et al., 2020), and because lower vaginal bacterial loads may affect the ability of RC urine to collect vaginal microbes, we separately evaluated the microbiota of paired RC urine and vaginal samples collected from peri/post-menopausal women. Though limited in sample size, our results suggest that paired mid-vaginal/RC urine samples in peri/post-menopausal women share similar community structure with kappa agreement values indicating substantial agreement, although CST concordance was just a bit lower than reproductive-age women.

### CST Discordance

Among all participants and sample types, we found 17.7% of urine samples were discordant for CST with the paired vaginal sample, including 11% of samples from reproductive-age women with RC urine (10/91), 13% of samples from reproductive-age women with CC urine (13/99), and 23% of samples from peri/post-menopausal women with RC urine (3/13). In reproductive-age women, discordance was found most often between CSTs III and IV [60% of discordant RC urine samples (6/10) and 23% of discordant CC urine samples (3/13)]. Still, urine samples assigned to CSTs III and IV were largely concordant with the CST assignment of the paired vaginal swab (84% for both). This is not surprising considering that *L. iners* is commonly found in a high relative abundance in CST IV, and that transitions are often observed between CST III and CST IV in longitudinal studies (Verstraelen et al., 2009; Gajer et al., 2012; Smith et al., 2012). Subtle differences in the relative abundance of *L. iners* affect assignment to CST III or CST IV, while overall community structures remain somewhat similar. Given that CST III samples often represent less optimal states compared to other *Lactobacillus-*dominated CSTs, future studies seeking to use urine samples as a proxy for the vaginal microbiota may consider categorizing CST III and CST IV samples together (92.6% concordant, reproductive-age women), rather than considering CST III with other *Lactobacillus-*dominated CSTs.

When considering the relative abundance of both shared and non-shared species using Yue-Clayton theta indices, our study identified strong similarities between paired RC and CC urine and vaginal microbiotas. ANCOM II did identify several key taxa as statistically likely to differ in their relative abundance, including *Gardnerella*, *Streptococcus, Corynebacterium* 1, and some species of *Prevotella*. However, it should be noted that ANCOM II defines significantly different taxa based on the strength of the evidence, and not the magnitude of the effect size. Among these significant taxa, we found small values for the centered log-ratio (CLR) mean difference, which represents the log-fold change in the relative abundance of that taxa relative to the geometric mean composition. Given the small paired differences in relative abundance shown in **Figure S4B**, we would not expect these differences to significantly impact CST assignment. While *Gardnerella* in particular is an important taxa when defining CST, less than 2% of paired vaginal and urine samples had absolute difference of 30% or more in their relative abundance of *Gardnerella*, which might explain some of the discordance between CST III and CST IV. However, it is important to note that the relative abundance of *Gardnerella* did not appear to be biased by sample type. Some urine samples had a higher relative abundance of *Gardnerella* compared to the paired vaginal sample, while other urine samples had a lower relative abundance. Consequently, we would not expect to observe any systematic differences in the relative abundance of *Gardnerella* in studies using urine samples. In addition, all of the top 25 most abundant taxa could be found in both urine and vaginal samples.

### Considerations for Clean-Catch Urine

The protocol for collecting a CC urine sample involves cleaning the labia and periurethral area before sample collection, and we had originally hypothesized that it might decrease the chance that urine collects vaginal microbes from the labia or introitus. It was noteworthy that both RC and CC urine samples demonstrated a similar bacterial composition to vaginal swabs. This finding could be explained by the urethra being colonized by similar organisms to the surrounding vulvovaginal environment, the proximity of the vaginal microbiota to the urethra, and the mechanical action of urine passing over the external vulva.

A high proportion of CC urine samples had fewer sequences than our quality control cutoff (19.5%; 24/123), compared to RC urine samples (0.8%; 1/128). This result may be attributed to an overall lower microbial burden in these samples due to the use of chlorhexidine wipes and collecting mid-stream urine. This observation is similar to another study utilizing CC urine that found approximately 14% of samples yielded undetectable bacterial DNA by 16S rRNA amplicon sequencing (Thomas-White et al., 2017). While our results suggest both RC and CC urine samples can be used as a proxy to study vaginal CSTs in reproductive-age women, RC urine may be favorable because of higher bacterial loads due to the contribution of vulvovaginal bacteria in the sample and not requiring mid-stream sampling and antibacterial wiping.

### Limitations and Future Directions

A set of three convenience studies were utilized, and DNA extraction and sequencing methodologies were not consistent between studies (**Table S6**). The differences in extraction methodologies between urine types make it challenging to directly compare rates of success in sequencing CC *versus* RC urine samples. These methodological differences may offer an explanation as to why samples from reproductive-age women with paired RC urine were dropped due to low sequence counts in the vaginal sample, while no samples from reproductive-age women with paired CC urine were dropped due to low sequence counts in the vaginal sample. We have previously reported that HiSeq, which was used for all urine samples from reproductive-age women and all vaginal samples from reproductive-age women with paired CC urine, produces greater mean quality scores and number of sequences per sample compared to MiSeq, which was used for a large proportion of the vaginal samples from reproductive-age women with paired RC urine (Holm et al., 2019). In a prior study, we reported complete within-subject agreement in vaginal CST assignment when comparing data sequenced on HiSeq and MiSeq Ilumina instruments (Holm et al., 2019), and so we do not expect the differences in sequencing methodologies to have any effect on CST assignment or concordance. Additionally, we have reported that VALENCIA CST assignments are also largely unaffected by choice of bioinformatic pipeline or variable region (France et al., 2020).

The sample size for peri/post-menopausal samples was limited, and therefore, we are unable to confirm whether the concordance between genitourinary and vaginal CSTs was lower in peri/post-menopausal women compared to reproductive-age women. Studies seeking to use urine as a surrogate for vaginal samples in peri/post-menopausal women may benefit from broadly categorizing the microbiota as *Lactobacillus-*dominated (CSTs I/II/III/V) *versus* low-*Lactobacillus* (CST IV), or as CSTs I/II/V *versus* CSTs III/IV (84.6% concordance for both).

It would also be of interest to evaluate the concordance between genitourinary and vaginal microbiota using other molecular techniques such as qPCR for the quantitative detection of specific species of interest, and to evaluate whether paired urine and vaginal samples share similar metagenomic profiles. Lastly, future studies could determine whether longitudinal profiles of the genitourinary microbiota reflect paired longitudinal profiles of the vaginal microbiota, and if personal behaviors, menstruation, or use of medications, such as hormonal contraception, affect the degree to which the composition of the genitourinary microbiota overlaps with the vaginal microbiota.

## Conclusions

Bacterial compositions of RC and CC urine samples demonstrated substantial agreement to paired mid-vaginal samples for both reproductive-age and peri/post-menopausal women. Urine samples may be a useful surrogate to evaluate broad community state type categories of microbiota, particularly for the purpose of hypothesis-generating research. Indeed, studies utilizing urine samples may provide important preliminary evidence to support conducting further research with vaginal samples.

## Data Availability Statement

The datasets are available at the National Center for Biotechnology Information (NCBI) Sequence Read Archive (SRA) BioProject accession number PRJNA208535 (recruited at University of Alabama at Birmingham), and the Database of Genotypes and Phenotypes (dbGaP) accession numbers phs.002169.v1.p1 (recruited at Johns Hopkins University; Hormonal Contraceptives Longitudinal Study) and phs.002211.v1.p1 (recruited at University of Maryland School of Medicine; Gynecology and Lubricant Effects Study).

## Ethics Statement

The studies were reviewed and approved by the Institutional Review Boards at the University of Alabama Birmingham, Johns Hopkins University School of Medicine and the University of Maryland Baltimore. The participants provided written informed consent.

## Author Contributions

SB: Conceptualization, formal analysis, and writing – original draft preparation. CR: Conceptualization, formal analysis, investigation, and writing – original draft preparation. MS: Formal analysis and writing – review and editing. JH: Data curation and writing – review and editing. JR: Funding acquisition, resources, and writing – review and editing. KG: Funding acquisition, resources, and writing – review and editing. RB: Conceptualization, funding acquisition, resources, and writing – review and editing. All authors contributed to the article and approved the submitted version.

## Funding

This work was funded by the National Institutes of Allergy and Infectious Diseases (NIAID) UH2-AI083264 (JR), R01-AI119012 (RB), and R01-AI089878 (KG). The funders had no role in study design, data collection, analysis, writing, or the decision to submit this manuscript.

## Conflict of Interest

JR is a co-founder of LUCA Biologics, a biotechnology company focusing on translating microbiome research into live biotherapeutic drugs for women’s health.

The remaining authors declare that the research was conducted in the absence of any commercial or financial relationships that could be construed as a potential conflict of interest.
