# Supplementary material for: Assessing the Concordance Between Urogenital and Vaginal Microbiota: Can Urine Specimens Be Used as a Proxy for Vaginal Samples?
Source: Front Cell Infect Microbiol. 2021 Jun 29;11:671413. doi: 10.3389/fcimb.2021.671413 (PMC8276069; doi:10.3389/fcimb.2021.671413)
Supplement: Supplementary file 1 [file DataSheet_1.docx]

**Table S1**: Mean and median sequence counts for paired urine and vaginal samples

|  |  |  | |
| --- | --- | --- | --- |
| **Project** | **Specimen type** | **Sequence Counts** | |
|  |  | *Mean* | *Median* |
| Reproductive-age women (N=91) | Vaginal | 17,693 | 6,093 |
|  | Random-catch urine | 49,178 | 50,152 |
| Reproductive-age women (N=99) | Vaginal | 62,554 | 55,420 |
|  | Clean-catch urine | 75,957 | 50,899 |
| Peri/post-menopausal women (N=13) | Vaginal | 78,869 | 54,717 |
|  | Random-catch urine | 49,048 | 56,409 |

**Table S2**: Concordance of random-catch urine to paired vaginal samples: Community State Type IV-A, IV-B and IV-C

|  | **Vaginal CST (Reproductive-age women)** | | | | | | |
| --- | --- | --- | --- | --- | --- | --- | --- |
|  | **I** | **II** | **III** | **IV-A** | **IV-B** | **IV-C** | **V** |
| **Urine CST** |  |  |  |  |  |  |  |
| **I** | **22** | 0 | 0 | 0 | 0 | 0 | 0 |
| **II** | 1 | **3** | 0 | 0 | 0 | 0 | 0 |
| **III** | 1 | 0 | **21** | 0 | 2 | 1 | 2 |
| **IV-A** | 0 | 0 | 0 | **0** | 1 | 1 | 0 |
| **IV-B** | 0 | 0 | 3 | 1 | **22** | 0 | 0 |
| **IV-C** | 0 | 0 | 0 | 1 | 2 | **4** | 0 |
| **V** | 0 | 0 | 0 | 0 | 0 | 0 | **3** |
|  |  | | | | | | |
|  | **Vaginal CST (Peri/Post-menopausal women)** | | | | | | |
|  | **I** | **II** | **III** | **IV-A** | **IV-B** | **IV-C** | **V** |
| **Urine CST** |  |  |  |  |  |  |  |
| **I** | **1** | 0 | 0 | 0 | 0 | 0 | 0 |
| **II** | 0 | **0** | 0 | 0 | 0 | 0 | 0 |
| **III** | 0 | 0 | **3** | 0 | 0 | 0 | 0 |
| **IV-A** | 0 | 0 | 0 | **0** | 0 | 0 | 0 |
| **IV-B** | 0 | 0 | 0 | 0 | **5** | 0 | 1 |
| **IV-C** | 0 | 0 | 0 | 0 | 0 | **1** | 0 |
| **V** | 0 | 1 | 0 | 0 | 0 | 1 | **0** |

**Table S3**: Concordance of clean-catch urine to paired vaginal samples: Community State Type IV-A, IV-B, IV-C

|  | **Vaginal CST** | | | | | | |
| --- | --- | --- | --- | --- | --- | --- | --- |
|  | **I** | **II** | **III** | **IV-A** | **IV-B** | **IV-C** | **V** |
| **Urine CST** |  |  |  |  |  |  |  |
| **I** | **41** | 0 | 4 | 0 | 0 | 0 | 0 |
| **II** | 0 | **2** | 0 | 0 | 0 | 0 | 0 |
| **III** | 1 | 1 | **21** | 0 | 0 | 0 | 0 |
| **IV-A** | 0 | 0 | 0 | **1** | 0 | 0 | 0 |
| **IV-B** | 0 | 0 | 3 | 4 | **9** | 0 | 1 |
| **IV-C** | 2 | 0 | 0 | 0 | 1 | **5** | 1 |
| **V** | 0 | 0 | 0 | 0 | 0 | 0 | **2** |

**Table S4:** Concordance of random-catch urine to paired vaginal samples: *Lactobacillus-*dominated (CSTs I/II/III/V) versus not *Lactobacillus-*dominated (CST IV)

|  | **Vaginal CST**  **Reproductive-age women** | |  |  | **Vaginal CST**  **Peri/post-menopausal women** | |
| --- | --- | --- | --- | --- | --- | --- |
|  | **I/II/III/V** | **IV** |  |  | **I/II/III/V** | **IV** |
| **Urine CST** |  |  |  |  |  |  |
| **I/II/III/V** | **53** | 3 |  |  | **5** | 1 |
| **IV** | 3 | **32** |  |  | 1 | **6** |

**Table S5:** Concordance of clean-catch urine to paired vaginal samples: *Lactobacillus-*dominated (CSTs I/II/III/V) versus not *Lactobacillus-*dominated (CST IV)

|  | **Vaginal CST** | |
| --- | --- | --- |
|  | **I/II/III/V** | **IV** |
| **Urine CST** |  |  |
| **I/II/III/V** | **72** | 0 |
| **IV** | 7 | **20** |

**Table S6**: DNA extraction methods and 16S rRNA gene amplicon sequencing platforms used in each study

|  |  | **Reproductive-age**  **women** | | **Peri/post-menopausal women** |
| --- | --- | --- | --- | --- |
|  |  | Paired RC urine | Paired CC urine | Paired RC urine |
| **DNA extraction:**  **vaginal** | MagAttract Microbial DNA kit | 1 | 4 | 0 |
|  | QS DSP Virus/Pathogen Midi Kit | 112 | 118 | 15 |
| **Sequencing platform:**  **vaginal** | Illumina MiSeq | 71 | 0 | 15 |
|  | Illumina HiSeq | 41 | 118 | 0 |
| **DNA extraction:**  **urine** | Quick-urine only | 112 | 0 | 0 |
|  | Quick-urine/  MagAttract | 0 | 118 | 15 |
| **Sequencing platform:**  **urine** | Illumina MiSeq | 0 | 0 | 15 |
|  | Illumina HiSeq | 112 | 118 | 0 |
