# Supplementary figures and images for: Assessing the Concordance Between Urogenital and Vaginal Microbiota: Can Urine Specimens Be Used as a Proxy for Vaginal Samples?
Source: Front Cell Infect Microbiol. 2021 Jun 29;11:671413. doi: 10.3389/fcimb.2021.671413 (PMC8276069; doi:10.3389/fcimb.2021.671413)

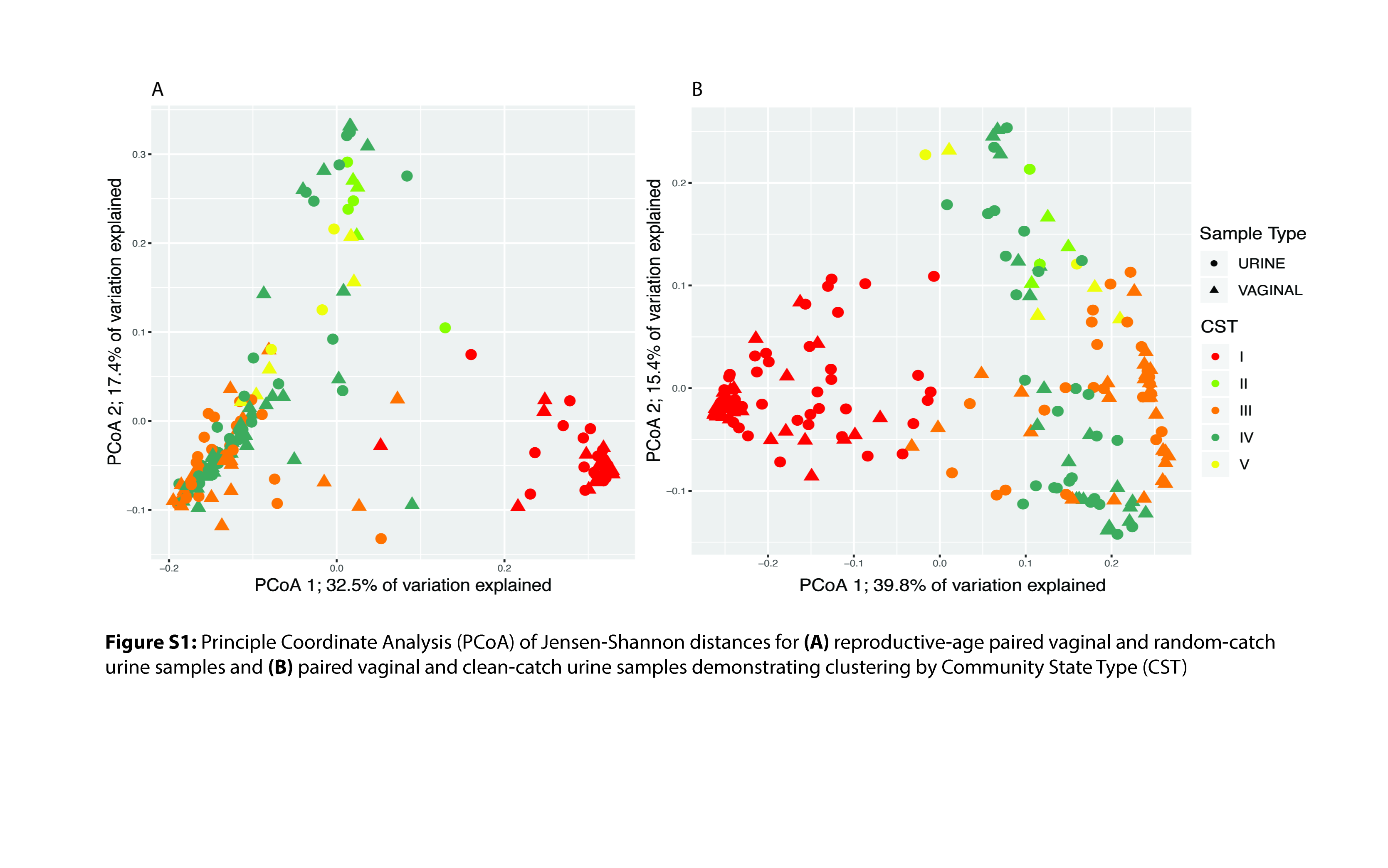

Supplement: Supplementary file 2 [file Image_1.tif]

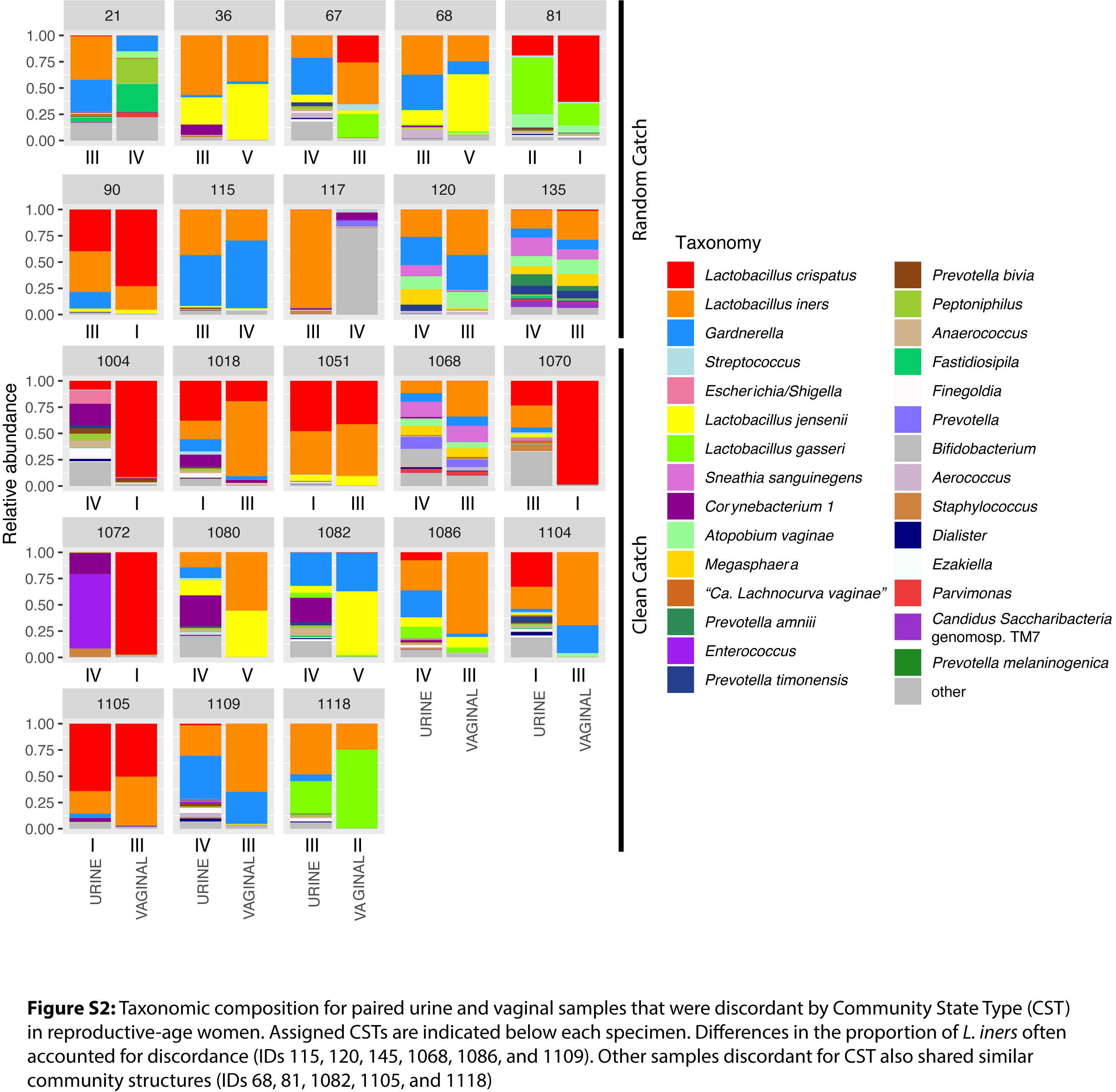

Supplement: Supplementary file 3 [file Image_2.tif]

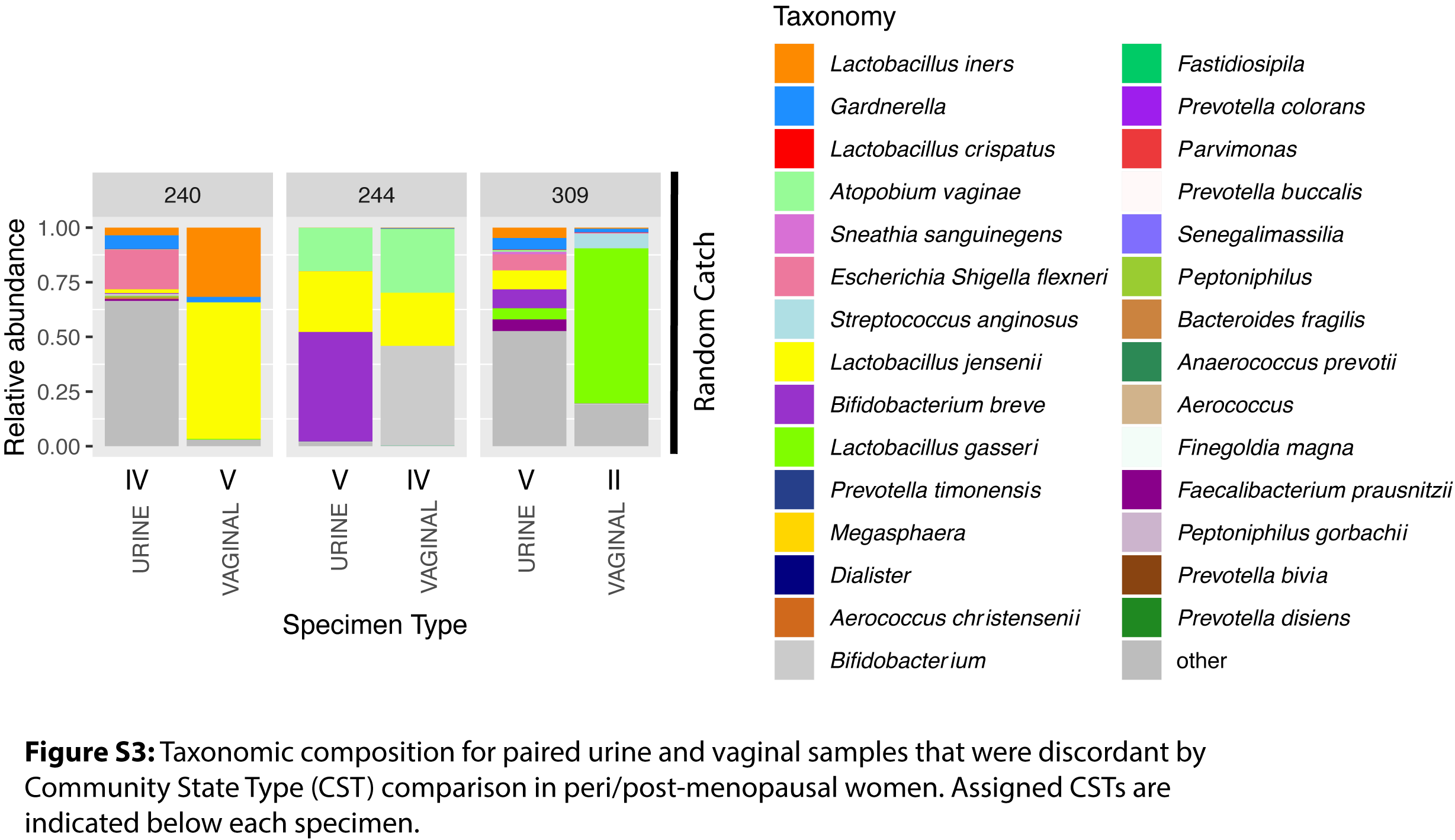

Supplement: Supplementary file 4 [file Image_3.tif]

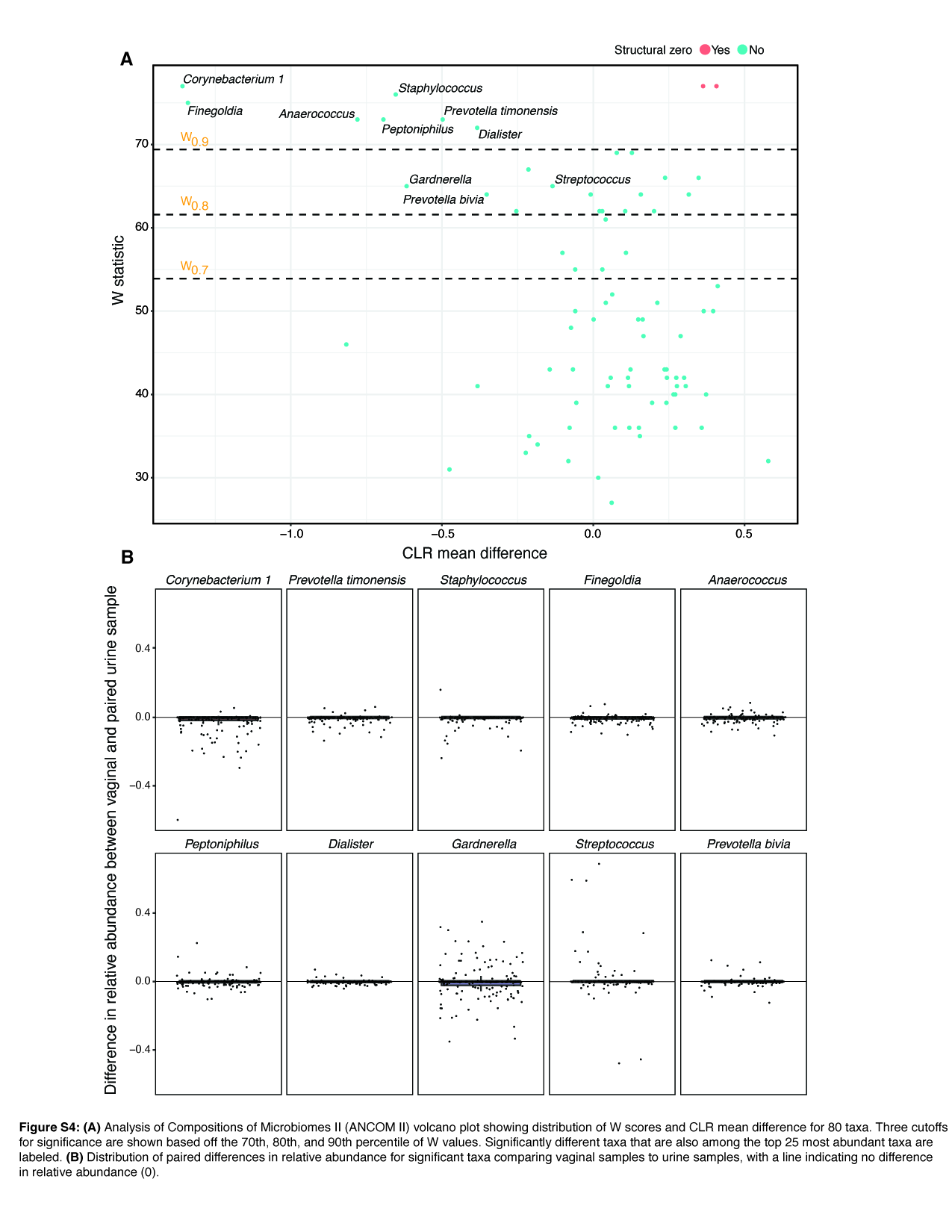

Supplement: Supplementary file 5 [file Image_4.tif]
